# Blood Morphology and Hematology of Adult Baikal Seals (*Pusa sibirica* Gmelin, 1788) Under Professional Care †

**DOI:** 10.3390/ani15020217

**Published:** 2025-01-15

**Authors:** Polina Esipova, Irina Suvorova, Veronika Yachmen, Igor Pushchin

**Affiliations:** 1 A.V. Zhirmunsky National Scientific Center of Marine Biology, Far Eastern Branch, Russian Academy of Sciences, St. Palchevskogo 17, 690041 Vladivostok, Russia; h.formula@inbox.ru (V.Y.); ipushchin@gmail.com (I.P.); 2 Center of Oceanography and Marine Biology “Moskvarium”, Building 23, Mira Avenue, 119, 129223 Moscow, Russia; i.suvorova@moskvarium.ru

**Keywords:** hematology, marine mammals, pinnipeds, true seals, blood cell morphology, *Pusa sibirica*

## Abstract

Studying the blood cell morphology of marine mammals provides an opportunity to elucidate the physiological mechanisms of adaptive changes associated with the aquatic habitat that occur at the cellular level, as well as adaptations to changing environmental conditions and under various physiological and pathological processes. The Baikal seal [*Pusa sibirica* (family Phocidae)] is endemic to the freshwater Lake Baikal, but comprehensive hematology data are not available. We studied the morphological features of blood cells of twelve clinically normal, adult Baikal seals (*n* = 6 males, *n* = 6 females) from two oceanariums under professional care for eight years. The morphology of mature and immature erythrocytes and inclusions are described. The blood of Baikal seals is characterized by the presence of erythrocytes with a size of 8.2 ± 0.6 µm; Howell–Jolly bodies were rarely observed, the number of reticulocytes ranged from 4.1 to 93.1 × 10^9^/L, and nucleated erythrocytes were absent. The morphological features of neutrophils, eosinophils, basophils, and platelets were described. Inter-individual and sex differences in the counts of basophils, platelets, red blood cells, and levels of hemoglobin, the mean corpuscular volume, and the mean corpuscular hemoglobin concentration were statistically observed. The results could be useful for Baikal seal veterinary care, immune response research, and comparative studies with other pinnipeds.

## Simple Summary

This study focuses on the hematological and morphological characteristics of blood cells from Baikal seals (*Pusa sibirica*) housed in two oceanariums over eight years. We described the morphology of various blood cells, including erythrocytes, leukocytes, and platelets. Additionally, we investigated the differences in blood parameters among sexes and individual seals. The data obtained can be used in marine mammal veterinary medicine as well as in scientific research for comparison of blood parameters in pinnipeds.

## 1. Introduction

Human-induced changes to marine and freshwater ecosystems, along with global climate change, are altering the environment [1], which affects the susceptibility of marine animals to infectious diseases, their lifespan, and their reproductive potential [2]. Marine mammals are indicators of aquatic ecosystem health, requiring monitoring and conservation due to various stressors [3,4,5]. The emergence of new pathogens in aquatic environments due to anthropogenic activities can significantly affect the health of marine animals, especially those living near humans [6,7].

Early detection of the initial signs of adverse animal health primarily occurs at the molecular and cellular levels. Therefore, the most accessible and informative sample for analysis is peripheral blood. Changes in the qualitative and quantitative parameters of blood can indicate pathological processes occurring in various organs and tissues [8,9,10,11,12,13]. A comprehensive analysis of the morphology of blood cells can provide insights into the effects of disease. This analysis encompasses various parameters, including the degree of cell activation, the presence of left shifts (indicative of an increase in immature cells, typically neutrophils, suggesting an inflammatory response), and abnormalities in cell morphology. The erythroid regenerative response (i.e., polychromasia) and the count of metarubricytes (nucleated red blood cells; nRBCs) are significant indicators of health [14]. Leukocyte differential count, hemoglobin and hematocrit levels, along with other blood cell characteristics, can provide crucial information for assessing an animal’s health and monitoring its condition during treatment [13,15].

Data on the blood cell morphology of healthy cetaceans and pinnipeds of various ages are limited and almost nonexistent for sick animals. Stacy and Nollens (2022) provided the most comprehensive range of information on the normal and abnormal morphology of blood cells in cetaceans, including the bottlenose dolphin (*Tursiops truncatus*), the killer whale (*Orcinus orca*), the beluga whale (*Delphinapterus leucas*), the Atlantic spotted dolphin (*Stenella frontalis*), and the long-finned pilot whale (*Globicephala melas*), and pinnipeds, including the monk seal (*Neomonachus schauinslandi*), the harbor seal (*Phoca vitulina*), the northern fur seal (*Callorhinus ursinus*), the Guadalupe fur seal (*Arctocephalus townsendi*), and the Steller sea lion (*Eumetopias jubatus*). A mild degree of polychromasia has been observed in pinnipeds, but it is minimal or absent in clinically normal cetaceans, where Howell–Jolly bodies may be present in erythrocytes. The leukocytes exhibit variations in cytoplasmic granularity and transparency, as well as differences in the size, presence, and heterogeneity of their granules. Unlike the fine, dust-like granules found in some cetaceans, pinniped neutrophils exhibit a transparent cytoplasm. The presence of atypical or reactive lymphocytes and plasma cells is rarely observed in healthy marine mammals [13].

Clark et al. (2002) described the morphology of blood cells in various wild and captured species of eared seals: New Zealand fur seal (*Arctocephalus forsteri*), subantarctic fur seal (*Arctocephalus tropicalis*), Australian fur seal (*Arctocephalus pusillus*), New Zealand sea lion (*Phocarctos hookeri*), and Australian sea lion (*Neophoca cinerea*). The erythrocytes were 6–9 μm in size and had central pallor. Additionally, a small number of polychromatophilic erythrocytes were present. The authors noted that neutrophils exhibited a transparent cytoplasm and had three to five lobed nuclei. In various species of fur seals, eosinophils and basophils displayed unequal numbers, sizes, and staining characteristics of their granules [16].

A study examining the erythrocyte morphology of California sea lion (*Zalophus californianus*) pups from the San Benito Archipelago, born during the unusually high temperatures of 2014 and 2015, found the presence of erythrocytes with atypical morphology, as well as microcytes and polychromatic red blood cells (RBCs). This indicates active erythropoiesis in the pups, which is likely normal for this stage of development [2].

Research on the blood morphology of true seals (Phocidae), including clinical and biochemical blood parameters, is limited [9,10,13,17,18,19,20,21]. Studying blood parameters in wild animals presents several challenges, such as stress from capture, limited sampling, and the distance to laboratory facilities. However, establishing baseline values and monitoring these parameters over time can only be effectively achieved when the animals are under human care.

The Baikal seal (*P. sibirica*) is a unique freshwater mammal of Lake Baikal, and it is one of three freshwater pinniped species in the world [22].

In one study, data on hematological and biochemical parameters were collected from two Baikal seals over a period of 4 to 6 years [23]. Another study presented the cellular composition of peripheral blood from 14 young and adult wild Baikal seals, along with a morphological description of their blood cells [24]. However, the following elements were not included: data regarding mature and immature erythrocytes and platelets, detected cellular inclusions, artifacts, variations in blood parameters by sex, and clear, detailed microphotographs of all blood cells.

No serious diseases were found in Baikal seals at the “Primorsky Aquarium” Branch of the National Scientific Center of Marine Biology (Far Eastern Branch, Russian Academy of Sciences, Vladivostok, http://www.imb.dvo.ru/index.php/en/, accessed on 6 January 2025) or the Center of Oceanography and Marine Biology “Moskvarium” (Moscow, https://moskvarium.ru/, accessed on 6 January 2025). Only minor injuries to the cornea of the eye were reported. Research on animals housed in oceanariums over extended periods, along with specialized laboratory studies, enables us to determine normal baseline blood values and provides an accurate and comprehensive analysis of blood cell morphology. Each species of marine mammal has unique characteristics in the structure of their peripheral blood cells. However, detailed studies of Baikal seals have not yet been conducted.

The aim of this study was to investigate the blood cell morphology and hematological parameters of clinically healthy adult Baikal seals over time.

## 2. Materials and Methods

All animals were born in 2012 (*n* = 10) and 2016 (*n* = 2) in the wild. At the age of 1–2, the Baikal seals were captured and transferred to oceanariums. This study includes 2 females and 1 male from the “Primorsky Aquarium,” a branch of the A.V. Zhirmunsky National Scientific Center of Marine Biology, Far Eastern Branch, Russian Academy of Sciences (Vladivostok, Russia), as well as 4 females and 5 males from the Center of Oceanography and Marine Biology “Moskvarium” (Moscow, Russia).

In the “Primorsky Aquarium,” the seals were housed in a single pool with a total water surface area of 88 m^2^ and a depth of 2.4 m. The pool featured a filtration and disinfection system, maintaining the water temperature between 6 and 8 °C and the air temperature between 13 and 15 °C.

In the “Moskvarium,” the seals were housed in two separate pools, with a total water surface area of 70 m^2^ and a rookery covering 24.2 m^2^. This facility utilized two filtration and cooling systems, keeping the water temperature between 3 and 5 °C and the air temperature between 14 and 17 °C.

In oceanariums, the diet of Baikal seals contains commercially available marine fish species. The diet included of greenling (*Pleurogrammus* spp.), chum salmon (*Oncorhynchus keta*), pink salmon (*Oncorhynchus gorbuscha*), and smelt (*Hypomesus* spp.) at the “Primorsky Aquarium.” The diet included capelin (*Mallotus* spp.), Pacific herring (*Clupea pallasii*), Baltic herring (*Clupea harengus membras*), and pink salmon (*Oncorhynchus gorbuscha*) at the “Moskvarium.”

No pregnant or lactating females were involved in the study, and no serious illnesses were reported.

The animal health assessment included daily inspections, behavioral observations, and an evaluation of food motivation. Ultrasonographic examinations and blood samples were analyzed every three months.

Blood samples were collected from the animals every three months throughout the year. The only treatment administered to the seals was Suprelorin (Virbac S.A., Milperra, NSW, Australia), given to male seals aged 9 years or older. Each male seal received either 4.7 mg or 9.4 mg of the drug once a year at the “Moskvarium.”

Blood samples were collected by veterinarians (*n* = 12) during routine wellness examinations from 2016 to 2023. The samples were obtained from the interdigital veins of the hind flippers using butterfly catheters (0.80 × 19 mm, 21G) and 10 mL syringes, and they were placed into EDTA tubes (Cerebrum, Shanghai, China). The samples were kept at a low temperature and sent for further analysis to the clinical laboratory at the “Primorsky Aquarium” (n = 118) or to the laboratory at Moskvarium (n = 270). A MEK 6550k analyzer (Nihon Kohden; Japan) was used at the “Primorsky Aquarium,” while the IDEXX ProCyte Dx analyzer (IDEXX Laboratories; Westbrook, ME, USA) was used at the “Moskvarium.” Blood samples were analyzed within one hour of being collected. The concentrations of total red blood cells (RBCs; 10^12^/L), white blood cells (WBC; 10⁹/L), platelets (PLTs; 10⁹/L), hemoglobin (HGB; g/L), hematocrit (HCT; %), mean corpuscular volume (MCV; fL), mean corpuscular hemoglobin (MCH; pg), and mean corpuscular hemoglobin concentration (MCHC; g/L) were determined using an automated method.

The concentrations of reticulocytes (Ret; %), total numbers of band neutrophils (Neut band; 10^9^/L), segmented neutrophils (Neut seg; 10^9^/L), eosinophils (Eos; 10^9^/L), basophils (Bas; 10^9^/L), monocytes (Mon; 10^9^/L), and lymphocytes (Lymph; 10^9^/L) were measured using a manual method. The Panchekov method was used to determine the erythrocyte sedimentation rate (ESR) [25].

Blood films were prepared on glass slides, fixed with May–Grunwald solution (BioVitrum, St. Petersburg, Russia), and stained with Romanovsky–Giemsa dye (MiniMed, Moscow, Russia) at room temperature (21 °C). This process was conducted to describe the morphology of the blood cells, including erythrocytes, platelets, and leukocytes, as well as to calculate the leukocyte count.

Supravital staining of erythrocytes was performed using brilliant cresyl blue (NPF ABRIS+, St. Petersburg, Russia). The samples were then prepared on slides and dried for the morphological description and quantification of reticulocytes.

All prepared blood films from two oceanariums were examined using a Carl Zeiss Axio Scope.A1 microscope (Carl Zeiss; Oberkochen, Germany), which was equipped with an AxioCam 105 Color digital camera (Carl Zeiss) and operated using ZEN 2.3 software (Carl Zeiss). A total of 388 blood samples from Baikal seals were analyzed. For each animal sample, at least 600 cells of each white blood cell type were measured, including erythrocytes, segmented neutrophils, monocytes, lymphocytes, eosinophils, and platelets. Additionally, 100 reticulocytes and band neutrophils, as well as 80 basophils, were counted in each sample.

### Statistical Analysis

The results are presented as the mean ± standard deviation of cell diameter measurements for each cell type. We assessed the statistical significance of differences in hematological analytes using the Kruskal–Wallis ANOVA on ranks. Post hoc Mann–Whitney U tests were used to reveal pairwise between-group differences [26]. Differences were considered significant at *p* ≤ 0.05, with a Bonferroni correction applied for the number of tests conducted. Comparisons of individual hematological analyses by sex were made only among five male and four female Baikal seals from the “Moskvarium.” Post hoc Mann–Whitney U tests were used to reveal differences between males and females.

In our study, we measured each blood parameter several times for each animal involved in the comparison. However, the samples compared came from different animals rather than from the same individual before and after treatment, as is the case with dependent samples. Therefore, we believe that using the Kruskal–Wallis test, along with post hoc pairwise tests, is a valid approach for comparing various blood parameters among different individuals, as in the present study.

All data were analyzed using GraphPad Prism 4.0 (GraphPad Software; La Jolla, CA, USA) and Microsoft Excel (Microsoft Corporation; Redmond, WA, USA).

## 3. Results

The blood of Baikal seals contains typical mammalian blood cells, including erythrocytes and leukocytes (neutrophils, lymphocytes, monocytes, eosinophils, and basophils).

### 3.1. Erythrocytes

**Figure 1:**
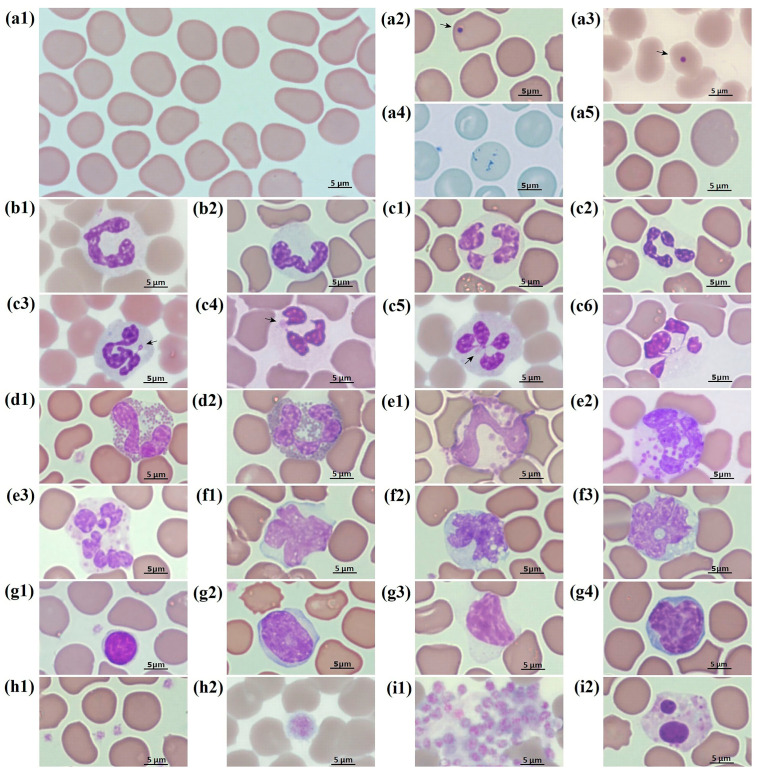
The morphology of peripheral blood cells of the Baikal seal (*Phoca sibirica*). (**a1**) Mature red blood cells; (**a2**,**a3**) mature erythrocyte with Howell–Jolly body (arrow); (**a4**) reticulocyte (cresyl diamond blue); (**a5**) polychromatophil; (**b1**,**b2**) band neutrophils; (**c1**) segmented neutrophil with 3 nucleus lobes connected by thin filaments of nucleoplasm sequentially; (**c2**) hypersegmented neutrophil with 5 nucleus lobes connected by sequential thin nuclear filaments; (**c3**) segmented neutrophil with Barr body (arrow); (**c4**) segmented neutrophil with 3 nuclear lobes connected by sequential thin nuclear filaments and nuclear vesicular appendages (blebs) (arrow); (**c5**) segmented neutrophil with 4 nuclear lobes connected by thin nuclear filaments from a central point and nuclear vesicular appendages (blebs) (arrow); (**c6**) hypersegmented neutrophil with 5 nuclear lobes connected by thin nuclear filaments; (**d1**,**d2**) eosinophils; (**e1**–**e3**) basophils; (**f1**–**f3**) monocytes with variable morphology; (**g1**) small lymphocyte; (**g2**) large lymphocyte; (**g3**) large lymphocyte; (**g4**) reactive (activated lymphocyte); (**h1**) platelets; (**h2**) large platelet; (**i1**) platelet aggregation (artifact); (**i2**) pyknosis of neutrophil nucleus in a fresh sample (presumed artifact). Romanowsky–Giemsa staining (unless otherwise noted); objective magnification ×100.

**Figure 2:**
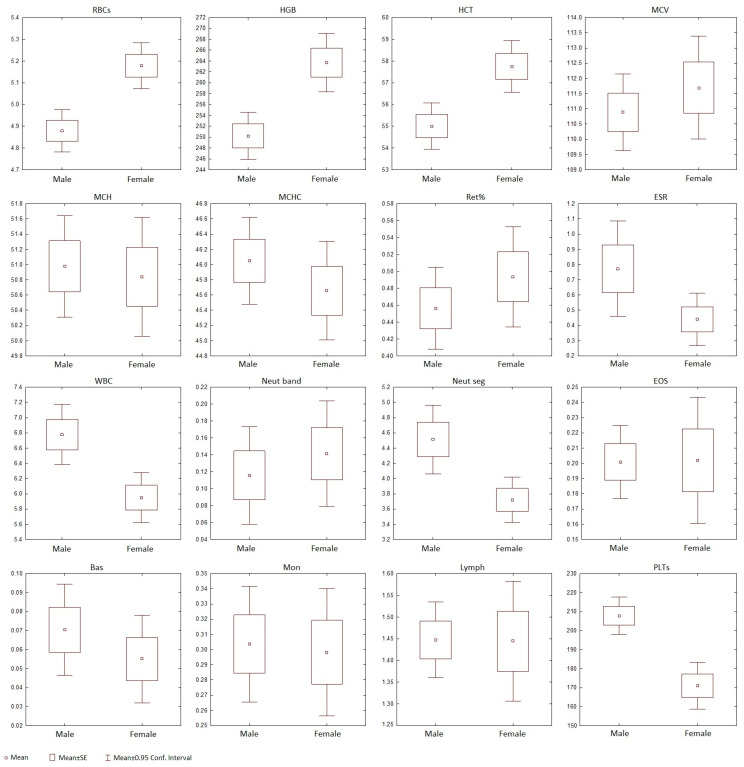
Means, standard errors of the mean, and 95% confidence intervals for original parameters characterizing blood analytes of male and female Baikal seals kept in the “Moskvarium”.

The erythrocytes of the Baikal seal included both mature and immature forms. The mature RBCs were disc-shaped and biconcave in structure, lacked a central pallor, and exhibited minimal size variation (Figure 1(a1–a5)). The average cell diameter was 8.2 ± 0.6 μm (Table 1). Howell–Jolly bodies, measuring approximately 1.0 ± 0.2 μm, were rarely observed in mature erythrocytes (Figure 1(a2,a3)). The proportion of these cells did not exceed 3% of the total number of RBCs. The concentration of RBCs, as well as the levels of HGB and HCT, were significantly higher in females kept in the “Moskvarium” compared to males (*p* = 0.000046; 0.000039; 0.000006, respectively) (Table 2 and Figure 2).

Reticulocytes were observed in the peripheral blood of clinically normal adult Baikal seals. These cells were identified by the presence of remnant ribosomes in the cytoplasm, which stained dark blue with brilliant cresyl blue (Figure 1(a4)). The average size of the reticulocytes was 8.8 ± 0.8 μm (Table 1). Additionally, polychromatic erythrocytes, likely reticulocytes, were noted in blood films stained with Romanovsky–Giemsa (Figure 1(a5)). The presence of metarubricytes in adult animals was recorded, albeit in very low numbers. Over the eight-year research period, both of the Baikal seals examined at the “Moskvarium” displayed a single metarubricyte in their blood films.

### 3.2. Leukocytes

**Figure 3:**
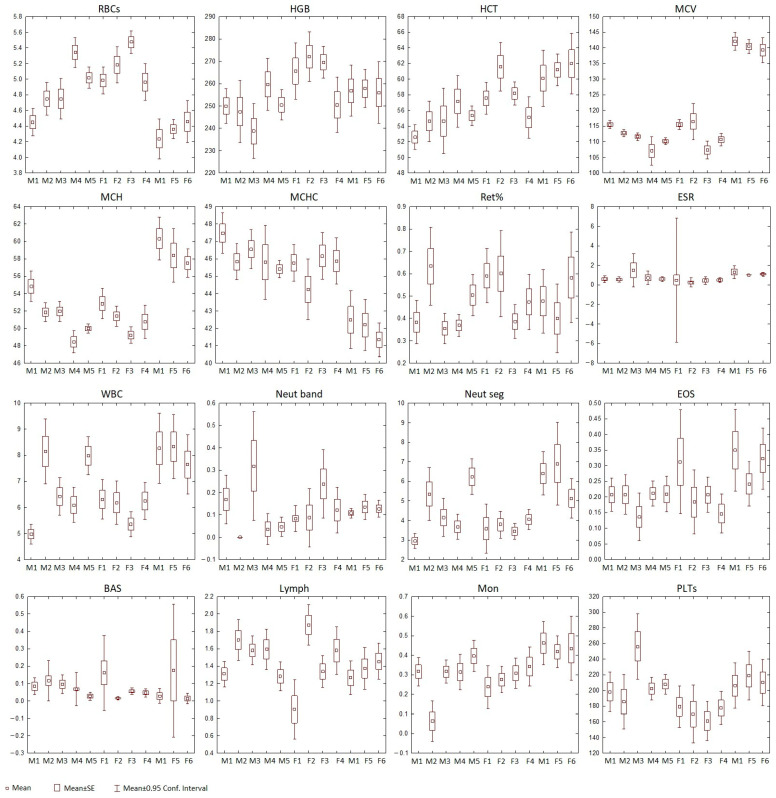
Means, standard errors of the mean, and 95% confidence intervals for the original parameters characterizing the blood analytes of the examined Baikal seal individuals.

The number of these cells varied among individuals (Table 3 and Table S1). However, no statistically significant differences were observed between the seals (Figure 3). Additionally, a comparison of the number of these cells in male and female Baikal seals from the “Moskvarium” also revealed no statistical differences (Table 2).

Neutrophils were the most abundant type of white blood cell found in Baikal seals. These cells were rounded and measured 12.8 ± 1.0 μm (Table 1). They had an almost transparent cytoplasm and exhibited pale pink, dusty granules (Figure 1(b1,b2,c1–c6)). Band neutrophils (Figure 1(b1,b2)) with a basophilic, free-lobed nucleus were observed in very low numbers (Table 4 and Table S1).

The nuclei of segmented neutrophils exhibited variations in shape and lobed segmentation based on their maturity. Older cells typically had a nucleus with five or more lobes. The lobes of the segmented neutrophil nuclei were connected by thin filaments of nucleoplasm either sequentially or emanating from one point (Figure 1(c1–c4)). A well-defined Barr body was observed only in the neutrophils of females (Figure 1(c3)). Both types of segmented neutrophils showed a lighter zone of decondensed chromatin (Figure 1(c4,c5)). The cytoplasm of segmented neutrophils was pale pink and contained dust-like pink and purple granules.

The diameter of eosinophils was larger than the neutrophils, measuring 14.5 ± 0.8 μm (Table 1). The nuclei of eosinophils typically had one or two lobes, although cells with multiple-lobed nuclei were also observed (Figure 1(d1,d2)). The chromatin displayed a range of staining, from dark purple (indicating more condensed regions) to bright purple (indicating less condensed regions). The pale blue cytoplasm of the eosinophils was filled with uniformly rounded pink granules, which had a diameter of 0.5 ± 0.1 μm (Figure 1(d1,d2)).

A small number of basophils was observed (Table 4 and Table S1). The mean diameter of the basophils was greater than that of the neutrophils and comparable to the eosinophils, measuring 14.9 ± 1.5 μm. (Table 1). The nuclei showed between 1 and 5 or more segments (Figure 1(e1–e3)), with the lobes connected by thin filaments of nucleoplasm sequentially. The cytoplasm was stained pale violet and contained a small number of large heterogeneous granules, averaging 0.8 ± 0.2 μm in size and varying in color intensity. 

### 3.3. Agranulocytes, Monocytes, and Lymphocytes

Monocytes were larger than neutrophils, averaging a diameter of 15.0 ± 1.6 μm (Table 1). The nuclei of monocytes were relatively large and bean-shaped, oval, or lobed. The chromatin was finely grained or lacy, with some areas of condensation. The cytoplasm was grayish blue and contained vacuoles of various sizes and shapes (Figure 1(f1–f3)).

The diameter of lymphocytes was significantly smaller than that of granulocytes and monocytes, measuring 9.6 ± 1.3 μm. Both large and small lymphocytes were observed, each exhibiting a round or slightly elongated oval nucleus (Figure 1(g1–g3)). The chromatin displayed smooth areas that alternated with denser, clumped formations. The nucleus occupied the largest portion of the cell, while the cytoplasm was relatively small and stained light blue or blue. Occasionally, activated lymphocytes were identified, characterized by irregular, tortuously shaped nuclei and looser chromatin, with their cytoplasm stained a deep blue-violet (Figure 1(g4)).

### 3.4. Platelets

The cytoplasm of the platelets was stained light blue and contained numerous small pink or purple granules (Figure 1(h1,h2)). The average diameter of the platelets was 2.1 ± 0.6 μm (Table 1), and macrothrombocytes, which are larger than 5 μm, were also identified (Figure 1 h2). The platelet count in the blood of males in the “Moskvarium” showed a higher count than that of females (*p* = 0.000023) (Table 2 and Figure 2). The most frequently observed artifact was platelet aggregation, which occurred due to their activation (Figure 1(i1)). Additionally, single nuclei and cells with pyknotic nuclei (Figure 1(i2)) were rarely observed in fresh blood films.

### 3.5. Statistical Analysis of Baikal Seals’ Blood Parameters

Our results revealed significant individual differences across all parameters, except for Ret and Eos (Figure 3 and Table 3). Consequently, these two parameters were excluded from further analysis. The overall pattern of pairwise differences varied significantly; some pairs differed in as many as six or seven parameters (for example, male 1 and male 6, male 2 and female 5, male 5 and female 3), while others showed no significant differences at all (such as male 1 and male 3, male 4 and female 2, and male 6 and female 5). The animals examined were quite similar in BAS, PLTs, and HGB, showing 0, 2, and 4 significant pairwise differences, respectively. In contrast, MCV, MCH, and RBCs showed the greatest variability among the animals, with 39, 28, and 25 significant pairwise differences, respectively (Figure 3).

Our results showed significant differences between the males and females kept in the “Moskvarium” in RBCs, HGB, HCT, and PLTs, suggesting that these parameters were sex-dependent (Figure 2 and Table 2).

## 4. Discussion

The current study provides a crucial baseline of blood parameters for captive Baikal seals. This baseline will facilitate the monitoring of changes in the analytes over time and assist in identifying potential effects of anthropogenic factors.

The study of blood parameters is important for assessing the physiological status of various animal species. It also helps to understand how individual blood parameters change with factors like age, sex, reproductive status, housing conditions, the development of pathologies [9,17,30], and the evolutionary adaptations of marine mammals to aquatic environments [31].

Baikal seals are particularly interesting as endemic representatives of freshwater Lake Baikal. This lake is known for its unique hydrological conditions, diverse fauna, and distinct microbiomes [3,31]. The morphology of the blood cells in Baikal seals was found to be similar to that of marine mammals, but with several morphological and quantitative peculiarities [13,28,29,32,33].

Mature Baikal seals’ RBCs have a similar structure to cetaceans’ and pinnipeds’; they lack nuclei, are biconcave disks, and are reddish when stained with standard cytology dyes [11,13]. The average RBC size of Baikal seals is slightly larger than that of harbor seals (*Phoca vitulina*) of the Commander Islands [27], spotted seals (our unpublished data), and bottlenose dolphins (*Tursiops truncatus*) [11,15,29] and close to that of beluga whales (*Delphinapterus leucas*) [13,28].

It is important to note that phocids show variations in their erythrocyte counts and their indices. The HGB and HCT levels of the Baikal seal, the northern elephant seal (*Mirounga angustirostris*), the ribbon seal (*Histriophoca fasciata*), the bearded seal (*Erignathus barbatus*), the ringed seal (*Pusa hispida*), and hooded seals (*Cystophora cristata*) are higher than those of the spotted seal, the harbor seal, and the gray seal (*Halichoerus grypus*). The MCV values of the northern elephant seal, the ribbon seal, the harp (*Phoca groenlandica*), hooded seals (*Cystophora cristata*), and bearded seal are higher compared to the Baikal seal and other phocids. The northern elephant seal is the only species among the phocids studied that exhibits low RBC counts. These variations in blood parameters may be related to the seals’ lifestyle, including their diving capabilities and evolutionary history [8,10,19].

High HCT levels are often correlated with diving depth, as demonstrated by the northern elephant seal. The unique environment of Lake Baikal, characterized by deep and cold waters with extended periods of ice cover, has led to specific adaptations in seals [34]. Furthermore, the elevated HCT and HGB levels observed in Baikal seals may reflect their unique habitat conditions. Prolonged immersion in cold water under ice without the ability to breathe for extended periods can lead to an increase in oxygen reserves, indicated by higher HCT and HGB levels.

The number of RBCs and the levels of HCT and HGB were higher in female Baikal seals at the “Moskvarium” compared to males. In contrast, the opposite tendency was observed in wild hooded seals, where males had higher levels than females. It is important to note that blood samples from hooded seals were collected during the breeding season, indicating that physiological differences related to sex may influence the results [8]. The adult Baikal seals at the “Moskvarium” lived in stable conditions, including consistent nutrition, no stress during medical procedures, no birthing events, and no significant health difficulties. These captive conditions possibly influence the blood parameters of both males and females compared to wild animals.

In this study, we found that the blood of adult Baikal seals, as well as adult harp and hooded seals [8], did not contain nucleated erythrocytes (metarubricytes). This contrasts with the blood of the walrus (*Odobenus rosmarus*) and cetaceans, such as bottlenose dolphins and beluga whales [18,19,28,33]. However, the presence of Howell–Jolly bodies (nuclear remnants in RBCs) and polychromatic RBCs (which contain remnant ribosomes in their cytoplasm) has also been observed in healthy beluga whales [28,33], as well as in terrestrial mammals, such as cats [15] and horses [35].

Marine mammal blood typically contains 1–5% Howell–Jolly bodies in normal RBCs [10,36]. The number of these nuclear remnants observed in Baikal seals was consistent with findings from other marine mammals and did not exceed the ranges reported in the literature.

Mature Baikal seal erythrocytes lack central pallor, as in most other marine mammal species [13,28,29,33,37], except for eared seals [16]. Slight anisocytosis, or variation in erythrocyte size, is commonly observed in the blood of Baikal seals. Reticulocytes were identified in blood films stained with cresyl diamond blue. The presence of polychromatophilic erythrocytes (likely reticulocytes) has been reported as normal in pinnipeds [13], and this is also applicable to adult Baikal seals.

The housing conditions of animals can influence their blood parameters. For instance, the concentration of RBCs in captured seals is lower than in wild seals, possibly due to the lack of deep-sea diving opportunities in captivity [38]. However, this has not yet been studied in Baikal seals.

Our findings regarding the distribution of leukocytes in the peripheral blood of Baikal seals are similar to those observed in some marine mammals [8,9,24,29,37,38]. The analysis revealed that segmented neutrophils were the predominant cell type, while band neutrophils were in low proportions among healthy individuals. The lymphocyte population mainly consisted of small cells. Additionally, we detected monocytes, eosinophils, and a minimal number of basophils.

The nuclei of the segmented neutrophils were typically three- to five-lobed. Their cytoplasm contains dust-like granules and is transparent for Baikal seals, similarly to many species of pinnipeds [13], it but differs from that of cetaceans [13,28]. There may be a difference in the enzyme composition of pinniped neutrophil cytoplasmic granules compared to cetaceans that are not detectable with Romanovsky–Giemsa dye. However, this hypothesis requires a more thorough investigation and the application of additional research methods.

This study identified two distinct types of segmented neutrophils. In these neutrophils, the lobes were connected by thin filaments of nucleoplasm either sequentially or emanating from one point. Similar types of segmented neutrophils were also found in the grey seal (*Halichoerus grypus*), the harp seal, and the bearded seal [39]. This suggests that these specific cells may be exclusive to certain species of true seals. It is currently unclear whether these cells differ in their functions. Nuclear vesicular appendages, known as blebs, have been observed in the neutrophils of other mammals [40]. While such blebs have occasionally been found in the segmented neutrophils of Baikal seals, their functions remain unknown, as well.

In this study, band neutrophils were rarely detected in the blood of Baikal seals, with an average count of 0.1 ± 0.1 × 10^9^/L. Juvenile Baikal seals exhibited even lower counts of these cells compared to adults [24]. These immature peripheral blood neutrophils usually appear to respond to acute infectious or inflammatory conditions [18]. Typically, immature peripheral blood neutrophils respond to acute infections or inflammatory conditions. However, the Baikal seals in this study were clinically healthy, which explains the anticipated low count of band neutrophils.

Eosinophils found in the blood of Baikal seals are morphologically similar to those in other marine mammals [13,28,29]. A detailed examination of these cells in pinnipeds revealed significant variations in granule size, density, and cytoplasm staining [13,27]. Increased levels of eosinophils are typically associated with the degree of parasite invasion, and their numbers can vary between captive and wild animals; they also depend on age [18,19]. However, this study found no difference in the number of eosinophils between young and adult wild Baikal seals [24].

Basophils are the least common type of cell found in the peripheral blood of marine mammals and may occasionally be absent [12,13,18,19,24,29,33,41,42]. In Baikal seals, basophils exhibited a reduced number of basophilic granules of varying sizes. The morphology of basophils differs among various species of pinnipeds, including the monk seal, the harbor seal, the northern fur seal, the Guadalupe fur seal, and the Steller sea lion [13]. The key distinguishing features of basophils in pinnipeds are their quantity, size, and density and the variability of their granules. However, the shape of the nucleus remains indistinct and chaotic in all of these animals. Basophils play a crucial role in defending against helminths, as well as in allergic reactions and autoimmune disorders [43]. In the presence of these pathological conditions, the concentration of basophils may increase. The low numbers of basophilic granulocytes observed in Baikal seals can be attributed to the fact that these individuals were free from parasites and in good health.

Monocytes in healthy Baikal seals display variations in shape, size, cytoplasmic vacuoles, and nuclear structure, similarly to other marine mammals [12,13,29].

Lymphocyte characteristics in pinnipeds are similar to other marine mammals, showing insignificant variation in cell size. The nuclear shape and cytoplasmic ratio vary depending on the specific type of lymphocyte [11,12,13,29].

Platelet size and anisocytosis are important diagnostic indicators [44]. In Baikal seals, the average platelet size was found to be 2.1 ± 0.6 μm, which is comparable to that of Irrawaddy dolphins [29]. Macrothrombocytes were rarely observed in blood films from Baikal seals, especially when compared to beluga whales [28]. The number of platelets in pinnipeds varies considerably across different studies. Specifically, among true seals, Baikal seals show a significantly lower platelet count than spotted seals, harbor seals, gray seals, and bearded seals but a higher count than ribbon seals [19].

Baikal seals have blood that is more viscous than that of some other marine mammal species [45], as indicated by a high hematocrit level. This increased viscosity can lead to artifacts in blood films, such as the formation of platelet aggregates. Therefore, it is important to consider these features to avoid mistaking them for symptoms of any disease.

The findings presented significantly enhance our understanding of Baikal seals’ physiology, specifically regarding blood cell morphology, leukocyte differential count, hemoglobin, and hematocrit levels. These characteristics are distinct from those of other pinniped species, reflecting their evolutionary adaptation to freshwater environments.

## 5. Conclusions

This study establishes a baseline database for blood morphology and hematology in Baikal seals based on a small group of managed-care animals observed over a multi-year period. Comparing blood parameters between wild and captive populations can help indicate the effects of human-induced factors on marine mammals, such as pollution, stress, infection, and poisoning. Additionally, it can reflect the characteristics of animals that have adapted to life in captivity. The blood cells of the Baikal seal exhibit several distinct characteristics. These include the large size of erythrocytes, a lack of central pallor, and the presence of Howell–Jolly bodies in mature erythrocytes. Additionally, the neutrophils have a transparent cytoplasm and two different types of segmented neutrophils. The relatively large size of the erythrocytes, along with elevated levels of HGB and HCT, may be due to the seal’s lifestyle and suggest an evolutionary adaptation to the cold and icy environment of freshwater Lake Baikal.

## Figures and Tables

**Table 1:** Blood cell diameters for Baikal seal and other marine mammals, μm.

Cell Type	Baikal Seal (This Study, Mean ± SD)	Harbor Seal [27]	Spotted Seal (Our Unpublished Data) (Mean ± SD)	Beluga Whale [28] (Mean ± SD)	Pacific Bottlenose Dolphins (Our Data) (Mean ± SD)	Irrawaddy River Dolphin [29] (Min–Max)
Metarubricytes (normoblasts)	ND *	ND	ND	9.9 ± 0.8	ND	ND
Reticulocytes	8.8 ± 0.8	9.3 ± 0.6	7.7 ± 0.6	10.3 ± 0.8	7.6 ± 0.5	ND
Red blood cells	8.2 ± 0.6	7.2 ± 0.7	7.6 ± 0.3	8.6 ± 0.7	6.8 ± 0.9	6–7
Neutrophils	12.8 ± 1.0	12.6 ± 0.8	12.3 ± 0.8	14.1 ± 1.1	13.1 ± 1.2	10–12
Eosinophils	14.5 ± 0.8	14.3 ± 0.8	13.3 ± 0.8	14.0 ± 1.2	12.3 ± 1.0	8–10
Eosinophil granules	0.5 ± 0.1	0.5 ± 0.1	0.4 ± 0.1	ND	0.5 ± 0.07	ND
Basophils	14.9 ± 1.5	13.9 ± 1.1	15.2 ± 1.1	13.0 ± 1.9	12.7 ± 0.2	ND
Basophil granules	0.8 ± 0.2	0.8 ± 0.3	0.9 ± 0.3	ND	0.5 ± 0.08	ND
Monocytes	15.0 ± 1.6	14.8 ± 1.2	15.5 ± 1.2	15.0 ± 2.2	13.9 ± 1.6	14–16
Lymphocytes	9.6 ± 1.3	9.0 ± 0.5 small; 11.3 ± 1.1 large	9.1 ± 1.4	10.8 ± 1.3	11.1 ± 1.6	8–10
Platelets	2.1 ± 0.6	2.6 ± 0.8	2.2 ± 0.7	3.5 ± 1.7	3.0 ± 0.7	1–2

* ND—no data.

**Table 2:** Mann–Whitney U tests comparing blood parameters of females and males kept in the “Moskvarium.” Parameters marked with asterisks differed significantly at *p* < 0.05000 (Bonferroni corrected).

Variable	Rank Sum Male	Rank Sum Female	U	Z	*p*-Value	Z Adjusted	*p*-Value	Valid N Male	Valid N Female
RBCs *	8271	8382	2600	−4.08	0.000	−4.07	0.000	106	76
HGB *	6416	7446	2138	−4.11	0.000	−4.11	0.000	92	74
HCT *	6496	7532	2031	−4.52	0.000	−4.51	0.000	94	73
MCV	4307	3319	1679	−0.80	0.422	−0.80	0.422	72	51
MCH	4543	3207	1842	−0.10	0.923	−0.09	0.923	73	51
MCHC	4784	2966	1640	1.12	0.262	1.12	0.261	73	51
Ret	3999	3023	1443	−1.24	0.215	−1.26	0.208	71	47
ESR	2963	953	628	1.47	0.141	1.58	0.115	63	25
WBC	10,485	5986	3136	2.41	0.016	2.42	0.015	106	75
Neut band	3540	2238	1125	−1.21	0.227	−1.29	0.195	69	38
Neut segm	6839	5251	2550	1.59	0.113	1.59	0.112	82	73
EOS	6635	5147	2519	1.45	0.147	1.45	0.147	81	72
BAS	2023	1464	798	0.44	0.659	0.45	0.654	47	36
Mon	8618	6782	3622	0.51	0.611	0.51	0.611	96	79
Lymph	6301	5635	2898	−0.19	0.845	−0.19	0.845	82	72
PLTs *	7698	3477	1586	4.24	0.000	4.24	0.000	88	61

**Table 3:** Kruskal–Wallis ANOVA by ranks tests comparing blood parameters of individual seals presently studied.

Variable	Pooled N	H	*p*-Value
RBCs	220	107	0.0000
HGB	204	304	0.0004
HCT	205	61	0.0000
MCV	161	126	0.0000
MCH	162	107	0.0000
MCHC	162	80	0.0000
Ret	156	25	0.0088
ESR	126	46	0.0000
WBC	219	77	0.0000
Neut band	145	49	0.0000
Neut segm	193	78	0.0000
EOS	191	27	0.0040
BAS	121	39	0.0000
Mon	213	44	0.0000
Lymph	192	46	0.0000
PLTs	187	34	0.0003

Note: Differences significant at *p* < 0.05 (Bonferroni corrected) were revealed for all parameters.

**Table 4:** The ranges and mean values of blood parameters for female and male Baikal seals housed at the “Moskvarium.”

		Males (n = 5, samples = 30)	Females (n = 4, samples = 25)
RBCs, ×10^12^/L	Mean ± st.dev *	4.8 ± 0.3	5.0 ± 0.2
	Range **	3.4–5.9	3.7–6.3
HGB, g/L	Mean ± st.dev	249.3 ± 7.0	264.4 ± 8.9
	Range	192.0–304.0	199.0–306.0
HCT, %	Mean ± st.dev	54.8 ± 1.5	58.0 ± 2.5
	Range	46.5–83.0	40.8–69.7
MCV, fl	Mean ± st.dev	111.4 ± 2.9	112.5 ± 3.9
	Range	99.1–139.7	102.4–133.6
MCH, pg	Mean ± st.dev	51.3 ± 2.2	51.0 ± 1.3
	Range	43.7–60.6	44.7–58.5
MCHC, g/L	Mean ± st.dev	46.1 ± 0.7	45.4 ± 0.7
	Range	34.7–54.9	39.0–52.0
Ret, %	Mean ± st.dev	0.4 ± 0.1	0.4 ± 0.1
	Range	0.1–1.1	0.2–1.0
ESR, mm/h	Mean ± st.dev	0.7 ± 0.4	0.4 ± 0.1
	Range	0.0–9.0	0.0–1.5
WBC, ×10^9^/L	Mean ± st.dev	6.6 ± 1.2	5.9 ± 0.4
	Range	4.0–12.1	3.0–8.8
Neut band, 10^9^/L	Mean ± st.dev	0.09 ± 0.1	0.1 ± 0.05
	Range	0.0–1.4	0.0–0.7
Neut seg, 10^9^/L	Mean ± st.dev	4.4 ± 1.2	3.6 ± 0.2
	Range	1.9–10.1	0.0–6.1
Eos, 10^9^/L	Mean ± st.dev	0.1 ± 0.04	0.1 ± 0.08
	Range	0.0–0.5	0.0–0.7
Bas, 10^9^/L	Mean ± st.dev	0.07 ± 0.02	0.05 ± 0.03
	Range	0.0–0.3	0.0–0.3
Mon, 10^9^/L	Mean ± st.dev	0.2 ± 0.1	0.2 ± 0.05
	Range	0.01–0.8	0.0–1.0
Lymph, 10^9^/L	Mean ± st.dev	1.4 ± 0.1	1.3 ± 0.3
	Range	0.6–2.8	0.0–3.0
PLTs, 10^9^/L	Mean ± st.dev	210.1 ± 25.4	171.8 ± 7.6
	Range	116.0–398.0	101.0–290.0

Mean ± st.dev *—mean and standard deviation; range **—minimum and maximum value.

## Data Availability

All original data are included in the Supplementary Materials.

## Supplementary Materials

The following supporting information can be downloaded at https://www.mdpi.com/article/10.3390/ani15020217/s1, Table S1. Physiological parameters and hematological analytes (ranges and mean values) of clinically normal, adult Baikal seals (*P. sibirica*) from two oceanariums under professional care.
