# Supplementary material for: Blood Morphology and Hematology of Adult Baikal Seals (Pusa sibirica Gmelin, 1788) Under Professional Care
Source: Animals (Basel). 2025 Jan 15;15(2):217. doi: 10.3390/ani15020217 (PMC11758329; doi:10.3390/ani15020217)
Supplement: Supplementary file 1 [file animals-15-00217-s001.zip › animals-3384622-supplementary.pdf]

Supplementary. Table S1. Physiological parameters and hematological analytes (ranges and mean values) of clinically normal, adult Baikal seals (*P. sibirica*) from two oceanariums under professional care.

		Male 1*	Male 2*	Male 3*	Male 4*	Male 5*	Female 1*	Female 2*	Female 3**	Female 4**	Male 6**	Female 5**	Female 6**
Body weight, kg		62.9 ±0.4	63.2 ±0.7	65.4 ±0.1	56.4 ±0.4	57.1 ±1.1	58.5 ±2.8	62.7 ±0.8	64.3 ±0.4	67.8 ±1.6	63.7 ±0.8	70.1 ±0.9	65.0 ±1.5
Body length, cm		127	129	143	137	142	136	138	132	132	135	122	120
Samples		21	19	23	20	30	18	21	25	22	20	20	19
RBCs, $\times10^{12}$/L	Mean±st.dev	4.4±0.3	4.7±0.3	4.7±0.5	5.3±0.4	5.0±0.3	4.9±0.2	5.1±0.4	5.4±0.3	4.9±0.5	4.2±0.4	4.3±0.2	4.4±0.4
	Range	3.4-5.1	4.0-5.2	3.5-5.8	4.4-5.9	4.3-5.8	4.3-5.3	4.3-5.7	4.4-6.3	3.7-5.6	3.3-4.9	3.9-4.6	3.7-4.9
HGB, g/L	Mean±st.dev	250.0±14.7	247.5±23.0	238.8±21.2	259.7±24.8	250.6±17.8	265.6±21.0	272.1±19.2	269.6±17.1	250.5±27.8	256.8±18.9	257.8±13.9	256.0±21.7
	Range	229-275	204.0-273.0	192.0-273.0	212.0-304.0	209.0-289.0	226.0-295.0	241.0-296.0	229.0-306.0	199.0-286.0	226.0-288.0	236.0-284.0	213.0-294.0
HCT, %	Mean±st.dev	52.6±3.1	54.6±4.2	54.6±7.2	57.1±7.0	55.3±3.3	57.5±3.3	61.6±5.1	58.1±3.6	55.0±5.9	60.1±5.9	61.1±3.3	61.9±6.0
	Range	46.5-58.6	47.1-58.8	47.7-75.2	47.8±83.0	47.0-60.5	49.6-62.0	53.6-69.7	48.1-66.7	40.8-63.4	49.3-73.0	56.8-66.5	52.4-73.4
MCV, fL	Mean±st.dev	115.5±2.0	112.7±1.6	111.6±1.6	107.1±8.8	110.2±2.5	115.5±2.4	116.5±7.4	107.4±5.3	110.7±3.6	142.1±4.7	140.4±3.7	139.3±6.3
	Range	113.4-119.8	110.6-115.6	109.2-114.7	99.1-139.7	104.3-114.1	110.0-119.2	105.8-133.6	102.4-126.5	102.8-115.3	136.6-148.8	135.4-148.2	132.0-149.8
MCH, pg	Mean±st.dev	54.8±2.7	51.8±1.6	51.9±1.5	48.4±2.4	49.9±1.2	52.8±2.6	51.4±1.5	49.2±1.7	50.7±3.4	60.3±4.1	58.3±5.0	57.5±2.5
	Range	51.9-60.6	49.0-54.8	50.1-54.6	43.7-55.0	47.7-54.1	49.0-58.5	49.0-53.4	45.4-52.0	44.7-58.1	52.9-68.8	44.5-66.8	57.4-61.7
MCHC, g/L	Mean±st.dev	47.4±1.8	45.8±1.6	46.5±1.4	45.8±4.1	45.4±1.1	45.7±1.5	44.2±2.2	46.1±2.5	45.8±2.3	42.5±2.7	42.1±2.4	41.3±1.5
	Range	45.6-51.9	43.1-48.5	44.7-48.9	34.7-54.9	43.5-47.9	44.0-49.6	39.3-47.9	39.0-49.5	43.5-52.0	35.6-46.6	36.6-46.8	38.0-43.4
Ret, %	Mean±st.dev	0.3±0.1	0.6±0.2	0.3±0.08	0.3±0.09	0.5±0.2	0.5±0.1	0.6±0.2	0.3±0.1	0.4±0.2	0.4±0.2	0.4±0.2	0.5±0.3
	Range	0.1-0.6	0.2-0.1	0.2-0.5	0.2-0.6	0.2-1.0	0.4-1.0	0.4-0.9	0.2-0.6	0.2-1.0	0.1-0.9	0.0-0.8	0.3-1.2
ESR, mm/h	Mean±st.dev	0.5±0.4	0.5±0.3	1.5±2.5	0.7±1.1	0.5±0.5	0.5±0.5	0.2±0.2	0.4±0.5	0.5±0.3	1.3±1.1	1.0±0.0	1.0±0.2
	Range	0.0-2.0	0.0-1.0	0.0-9.0	0.0-4.6	0.0-1.5	0.0-1.0	0.0-0.5	0.0-1.5	0.0-1.0	1.0-5.0	1.0-1.0	1.0-2.0
WBC, $\times10^{9}$/L	Mean±st.dev	4.9±0.8	8.1±2.3	6.4±1.5	6.0±1.4	7.9±1.9	6.3±1.2	6.1±1.4	5.3±1.1	6.2±1.5	8.2±2.2	8.3±2.0	7.6±1.7

	Range	4.0-7.4	5.2-13.6	4.2-9.7	4.3-9.5	4.8-12.1	4.2-7.9	3.6-7.9	4.1-8.4	3.0-8.8	5.8-13.3	5.9-12.6	5.3-11.9
Neut band, 10^9/L	Mean±st.dev	0.1±0.1	0.0±0.0	0.3±0.4	0.03±0.1	0.04±0.08	0.08±0.02	0.08±0.1	0.2±0.2	0.1±0.1	0.1±0.03	0.1±0.09	0.1±0.05
	Range	0.0-0.5	0.0-0.0	0.0-1.4	0.0-0.4	0.0-0.3	0.06-0.1	0.0-0.4	0.0-0.7	0.0-0.6	0.06-0.1	0.06-0.3	0.05-0.2
Neut seg, 10^9/L	Mean±st.dev	2.9±0.8	5.3±2.1	4.1±1.2	3.6±1.2	6.2±2.1	3.5±1.9	3.7±1.2	3.4±0.9	4.0±1.1	6.4±1.8	6.9±3.5	5.1±1.5
	Range	1.9-5.3	2.7-9.6	2.6-6.4	2.3-7.3	3.0-10.1	0.0-5.7	1.5-5.7	2.1-6.1	2.0-5.8	4.7-10.1	3.7-16.3	2.6-8.6
Eos, 10^9/L	Mean±st.dev	0.2±0.1	0.2±0.09	0.1±0.09	0.2±0.07	0.2±0.1	0.3±0.2	0.1±0.1	0.2±0.1	0.1±0.1	0.3±0.2	0.2±0.1	0.3±0.1
	Range	0.0-0.4	0.03-0.3	0.02-0.2	0.07-0.3	0.0-0.5	0.0-0.7	0.05-0.7	0.04-0.5	0.0-0.5	0.07-0.7	0.06-0.4	0.06-0.6
Bas, 10^9/L	Mean±st.dev	0.08±0.1	0.1±0.04	0.09±0.06	0.06±0.01	0.02±0.04	0.1±0.1	0.01±0.01	0.05±0.01	0.04±0.04	0.02±0.06	0.1±0.6	0.01±0.04
	Range	0.0-0.3	0.06-0.1	0.0-0.2	0.0-0.07	0.0-0.1	0.06-0.3	0.0-0.02	0.04-0.08	0.0-0.1	0.0-0.2	0.0-2.2	0.0-0.1
Mon, 10^9/L	Mean±st.dev	0.3±0.1	0.06±0.1	0.3±0.1	0.3±0.1	0.3±0.1	0.2±0.1	0.2±0.1	0.3±0.1	0.3±0.2	0.4±0.1	0.4±0.1	0.4±0.2
	Range	0.05-0.5	0.01-0.5	0.1-0.5	0.1-0.8	0.1-0.8	0.0-0.5	0.07-0.5	0.04-0.9	0.04-1.0	0.1-0.7	0.1-0.6	0.1-1.0
Lymph, 10^9/L	Mean±st.dev	1.3±0.3	1.7±0.3	1.5±0.2	1.5±0.4	1.2±0.3	0.9±0.5	1.8±0.3	1.3±0.4	1.5±0.6	1.2±0.3	1.3±0.3	1.4±0.3
	Range	0.7-2.1	1.0-2.3	1.2-2.0	0.6-2.8	0.7-2.2	0.0-1.9	1.4-2.9	0.4-2.2	0.4-3.0	0.8-1.7	0.6-1.9	0.9-2.0
PLTs, 10^9/L	Mean±st.dev	198.4±44.3	185.7±56.1	256.2±60.4	202.3±30.9	207.9±32.5	179.0±38.2	170.0±50.3	161.0±53.5	177.5±43.7	206.1±46.7	218.7±50.4	209.9±45.5
	Range	116.0-302.0	127.0-346.0	196.0-398.0	117.0-250.0	173.0-336.0	125.0-238.0	111.0-263.0	101.0-290.0	102.0-265.0	135.0-272.0	106.0-298.0	153.0-284.0

* Baikal seals kept in "Moskvarium", ** Baikal seals kept in "Primorsky Aquarium".
